# The bHLH transcription factor SPATULA regulates root growth by controlling the size of the root meristem

**DOI:** 10.1186/1471-2229-13-1

**Published:** 2013-01-02

**Authors:** Srilakshmi Makkena, Rebecca S Lamb

**Affiliations:** 1 Plant Cellular and Molecular Biology Graduate Program, The Ohio State University, Columbus, OH, USA; 2 Department of Molecular Genetics, The Ohio State University, Columbus, OH, USA

## Abstract

**Background:**

The *Arabidopsis thaliana* gene *SPATULA* (*SPT*), encoding a bHLH transcription factor, was originally identified for its role in pistil development. *SPT* is necessary for the growth and development of all carpel margin tissues including the style, stigma, septum and transmitting tract. Since then, it has been shown to have pleiotropic roles during development, including restricting the meristematic region of the leaf primordia and cotyledon expansion. Although *SPT* is expressed in roots, its role in this organ has not been investigated.

**Results:**

An analysis of embryo and root development showed that loss of *SPT* function causes an increase in quiescent center size in both the embryonic and postembryonic stem cell niches. In addition, root meristem size is larger due to increased division, which leads to a longer primary root. *spt* mutants exhibit other pleiotropic developmental phenotypes, including more flowers, shorter internodes and an extended flowering period. Genetic and molecular analysis suggests that *SPT* regulates cell proliferation in parallel to gibberellic acid as well as affecting auxin accumulation or transport.

**Conclusions:**

Our data suggest that *SPT* functions in growth control throughout sporophytic growth of Arabidopsis, but is not necessary for cell fate decisions except during carpel development. *SPT* functions independently of gibberellic acid during root development, but may play a role in regulating auxin transport or accumulation. Our data suggests that *SPT* plays a role in control of root growth, similar to its roles in above ground tissues.

## Background

The primary root of *Arabidopsis thaliana* has a simple and consistent organization of cell types [1]. Roots are divided into three distinct tissue zones along the proximal-distal axis. The most distal area is the zone of cell division or meristematic zone. A zone of cell elongation occurs just proximal to the division zone and the zone of cell differentiation or zone of maturation is the most proximal [2]. Within the root apical meristem (RAM), stem cells surround a group of four mitotically less active cells called the Quiescent Center (QC; [1]). The QC, together with its surrounding four types of stem cells (columella stem cells, epidermal/lateral root cap stem cells, cortex/endodermal stem cells and vascular stem cells), forms the stem cell niche [3]. The RAM is established during embryogenesis. In Arabidopsis, the zygote divides asymmetrically to form an apical and a basal daughter cell. Three rounds of stereotyped cell divisions in the apical daughter cell give rise to the apical and central regions of the embryo whereas the transverse divisions in the basal cell make about 6-9 cells that make up the extra-embryonic suspensor. The uppermost cell of the suspensor becomes the hypophysis, which divides transversely to make an upper and lower hypophyseal cell. The upper hypophyseal cell forms the QC and the lower hypophyseal cell forms the columella stem cells and the central root cap. The rest of the RAM arises from derivatives of the apical cell [3-5].

A complex network of transcription factors regulates specification of the root stem cell niche. The AP2/ERF transcription factor-encoding genes *PLETHORA1 (PLT1)* and *PLT2* are transcribed in response to auxin in the early basal embryo and are redundantly required for QC identity and stem cell maintenance [6]. Ectopic embryonic expression of *PLT1* and *PLT2* can induce the formation of a RAM, including the QC and initial cells [6]. The *PLT* genes are expressed in a gradient with maximal expression in the stem cell niche promoting stem cell identity and maintenance. Lower levels promote mitotic activity of stem cell daughter cells and low levels promote cell differentiation [7]. Two GRAS family transcription factors, SHORTROOT (SHR) and SCARECROW (SCR), regulate both radial patterning and stem cell niche specification in the root [8,9]. *SHR* is necessary both for the periclinal division of cortex/endodermal initials and endodermal specification [10-13]. *SCR* is required for the periclinal asymmetric division of the cortical/endodermal initial daughter cells and cell-autonomously required for QC identity [9,14,15].

The *SPATULA (SPT)* gene encodes a basic helix-loop-helix (bHLH) transcription factor and was originally identified for its role in carpel organogenesis [16]. *SPT* is necessary for the development and proliferation of the carpel margins and for development of tissues derived from the margin [16,17]. *SPT* is partially redundant with the closely related bHLH-encoding gene *ALCATRAZ (ALC)* and functions during fruit development to specify formation of the valve margin tissue in addition to margin tissues during pistil development. These proteins can heterodimerize and *SPT* can complement dehiscence defects in *alc* mutants if expressed appropriately, although the converse is not true [18]. In addition to its interaction with ALC, SPT interacts genetically and physically with another bHLH protein, INDEHISCENT (IND), in both carpel margin and fruit valve specification and these transcription factors may bind common target genes cooperatively [19]. SPT and IND proteins regulate auxin accumulation in the apical region of the developing carpels, important for carpel margin specification. This is due at least in part by their direct regulation of expression of two genes encoding members of the AGC3 family of protein kinases (*PID* and *WAG2*) that phosphorylate and control activity of PIN auxin efflux carriers [19].

Although *SPT* has been most extensively studied in the context of floral development, it has been shown to be involved in seed germination and leaf and cotyledon development [20-23]. *SPT* has also been shown to mediate vegetative growth repression in response to cool day temperatures [22] and *spt* mutants have larger leaves due to increased cell numbers and an enlarged meristematic region in leaf primordia [21] and larger cotyledons due to increased cell expansion [23]. Consistent with its broad function, *SPT* is expressed in proliferating regions of both vegetative and reproductive tissues, including the root [16,24].

Here, we investigate the role of *SPT* in root growth. *spt* mutants have longer primary roots due to an increase in cell proliferation. Examination of the RAM of these mutants showed that QC size is increased as is cell division in the initial cells, as measured by the cell division marker *pCYCB1;1::GUS*. The increased QC size arises in the embryo and extra divisions continue throughout root development. The effect of loss of *SPT* function on root growth is independent of GA but shares some common targets with this hormone. *spt* mutants show a larger auxin maximum at the root tip. Analysis of the mutants’ responses to exogenous auxin and auxin transport inhibitors suggests that auxin transport is likely to be altered. Control of auxin transport may be a common mechanism by which *SPT* regulates growth in the plant. Our results uncover the importance of *SPT* in the regulation of RAM size control.

## Results

### *SPT* is necessary for multiple developmental aspects of plant development

**Figure 1:**
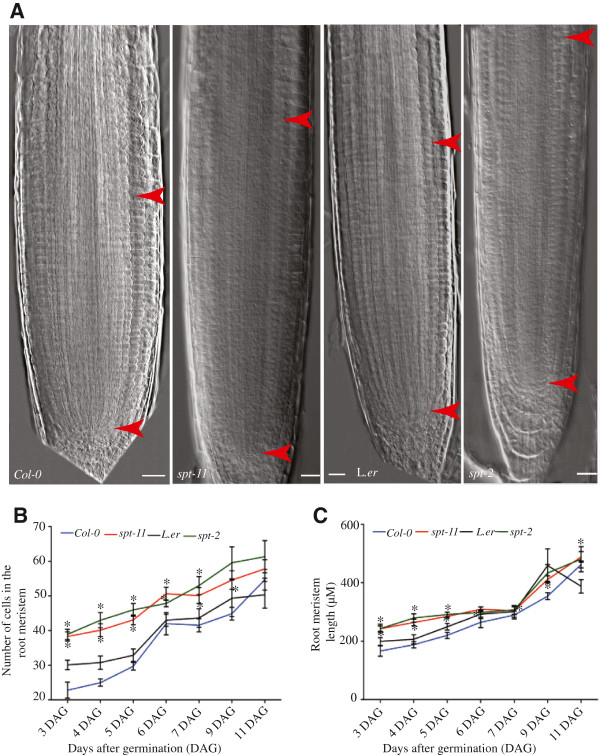
***spt *mutants have larger RAMs than wild type.** (**A**) Micrographs of 5 DAG roots. Arrowheads indicate the zone of cell division. (**B**) RAM size expressed as number of cells in the cortex. (**C**) Length of the RAM. Values are means (n = 15) ± standard error. Asterisks indicate values significantly different from wild type at P < 0.05. Scale bars indicate 100 μm.

**Figure 2:**
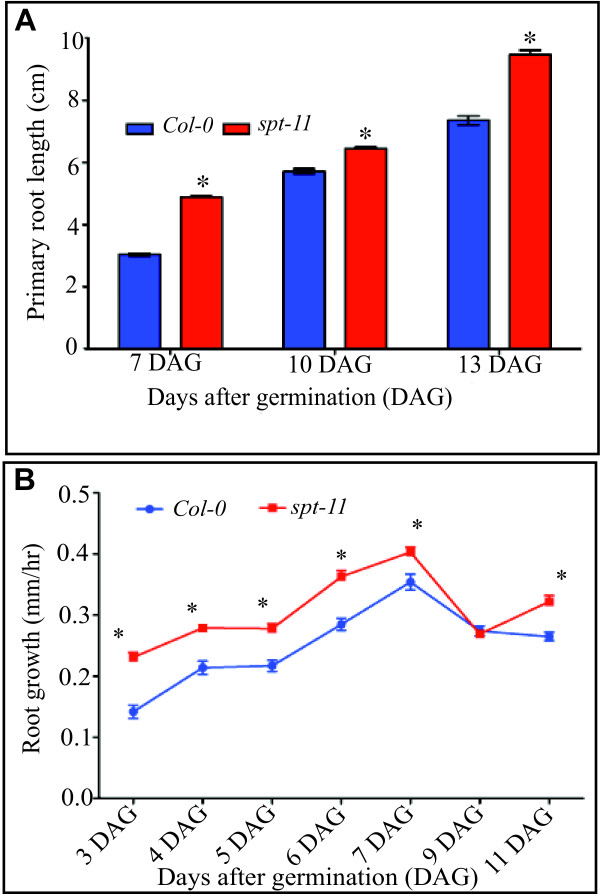
**Increased growth of *spt *roots.** (**A**) Primary root length (n = 90) at 7, 10 and 13 DAG. (**B**) Primary root growth rate (n = 30) expressed as mm/hr. Error bars indicate standard error. Asterisks indicate values significantly different from wild type at P < 0.05.

Although analysis of *SPT* function has been confined to the shoot, *SPT* is expressed in the root as well (Additional file 1; [24]). In order to analyze the possible function of *SPT* in this region, the root meristem of *spt-2* and *spt-11* mutants was compared to that of their respective wild types (Landsberg *erecta* (L*. er*) and Columbia-0 (Col-0)*.* Throughout the period of our observation, both *spt-2* and *spt-11* have longer root meristems (defined as the area between the QC and the first elongating cell in the root cortical layer; [25]) when compared to wild type, both as measured by number of cells and by length (Figure 1). Similar results were obtained when expression of the G2-M marker *CYCB1;1::GUS*[26] was observed*. spt-11* root meristems have a larger region of cells expressing this marker when compared to wild type (Additional file 2) and have more cells expressing this marker (an average of 41.3 cells per root vs. 35.2 cells per root in Col-0 (n = 15 seedlings). The primary root lengths of *spt-11* plants were longer than wild type (Figure 2A). A similar trend was seen in *spt-2* (data not shown); however, due to the tendency of roots to grow aslant and curl on vertical agar plates in the L. *er* background [27], which makes root measurements more difficult, this background was not as extensively analyzed. Primary root growth rates increase through 7 DAG and decline at 9 DAG, in both wild type and *spt-11* mutants, although the growth rate of *spt-11* roots was significantly higher than Col-0 (Figure 2B).

We also examined other growth parameters in *spt* mutants to determine how pleiotropic the function of this gene is. Both *spt-2* and *spt-11* plants are taller than wild type with more flowers. However, the internodes of these plants are shorter (Table 1). *spt-11* plants flowered earlier than Col-0*,* measured both as number of days to flower and number of leaves (Table 1). In contrast, *spt-2* plants flowered significantly later than L*. er*, suggesting that accession specific modifiers of *SPT* function may exist. Alternatively, the differences in flowering time could be due to the nature of the *SPT* alleles. *spt-11* is a knock down allele caused by a Ds insertion [21] while *spt-2* results from a missense mutation that changes an arginine to a lysine within the basic domain, a change that has been shown to abolish DNA binding in other bHLH proteins [16]. The differences in the nature of the alleles may contribute to the differences seen in flowering time in addition to or instead of the strain background.

**Table 1:** ***SPT *** **impacts plant growth**^**a**^

**Genotype**	**Flowering time**		**Inflorescence stem**			**Total plant height**^**b**^
	**Days to flower**	**Number of rosette leaves**	**Number of flowers**	**Number of internodes**	**Length of internodes**^**b**^	
L. *er*	22.4 ± 0.4	17.6 ± 0.6	49.1 ± 1.0	51.9 ± 1.0	0.57 ± 0.01	30.0 ± 0.7
*spt-2*	23.6 ± 0.3^c^	27.1 ± 0.9^c^	74.5 ± 1.2^c^	78.0 ± 1.3^c^	0.42 ± 0.004^c^	35.0 ± 0.5^c^
Col-0	29.9 ± 0.7	39.3 ± 1.4	71.7 ± 2.0	75.0 ± 1.9	0.83 ± 0.008	63.2 ± 1.6
*spt-11*	28 ± 0.8	36.8 ± 1.5	80.8 ± 2.8^c^	84.0 ± 2.6^c^	0.77 ± 0.012^c^	65.8 ± 1.3

Values are means ± standard error. ^a^All plants grown together. ^b^Measured in centimeters. ^c^Values significantly different from wild type at P < 0.05.

### *SPT* controls the size of the quiescent center of both the embryonic and postembryonic stem cell niches

**Figure 3:**
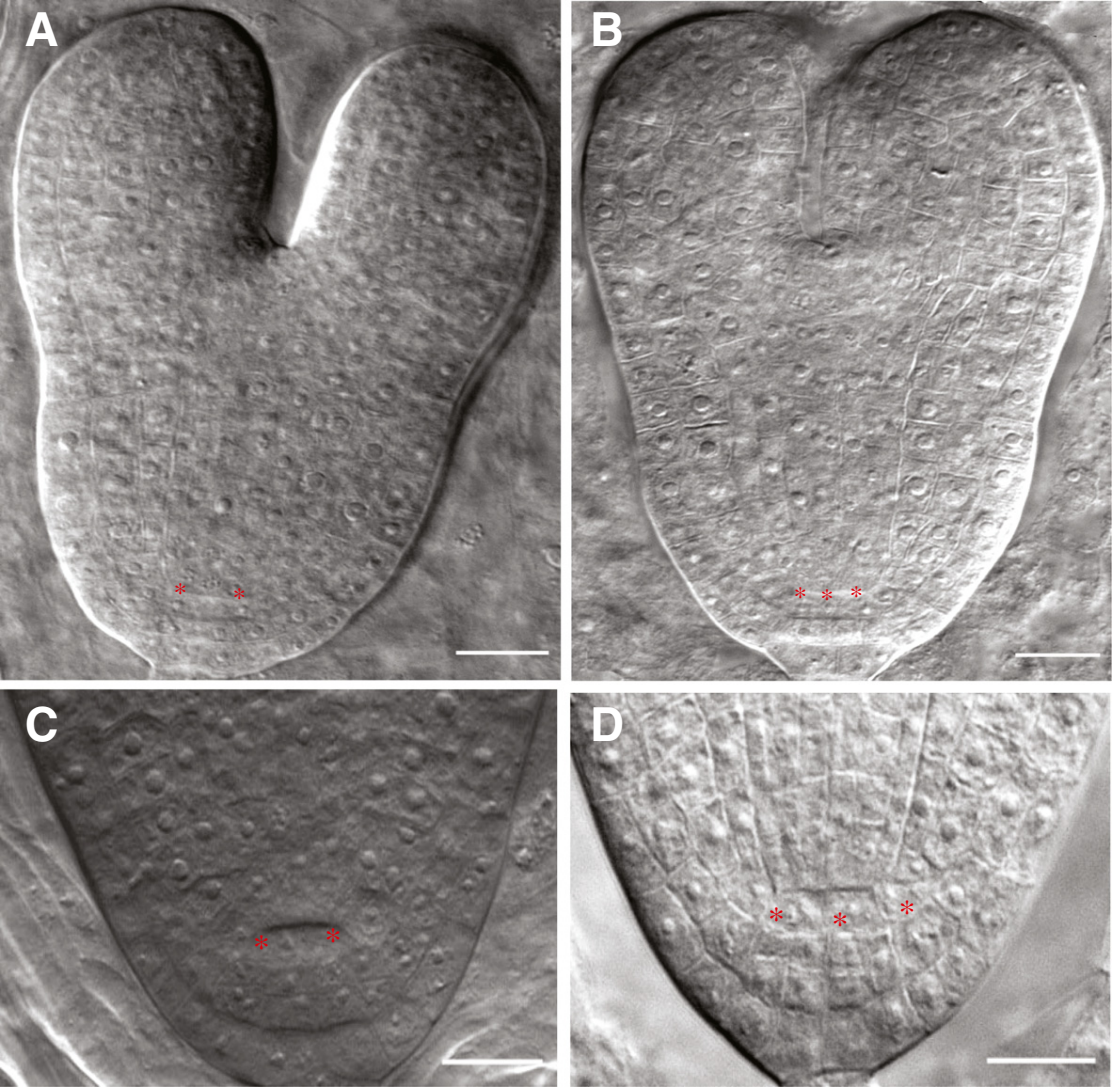
**QC size is increased in *spt-11 *embryos.** (**A-D**) Micrographs of embryos. Asterisks indicate cells in the QC. (**A, C**) Col-0*.* (**B, D**) *spt-11*. Scale bars indicate 50 μm.

We have investigated stem cell organization and number in *spt* mutants during the embryonic and postembryonic stages. Our observations of embryos indicate that there is no difference in the number of QC progenitor cells (data not shown) between wild type and *spt-11*, whereas the number of QC cells in *spt-11* differs from Col-0 at later stages of embryo development starting at the late heart stage (Figure 3B, D). In wild type, 90% of the observed embryos (n = 30) had four QC cells in both torpedo and mature embryo stages while the other 10% had 6 cells. In *spt-11* embryos, only 57% of the torpedo stage (n = 35) and 51.5% of the mature embryos (n = 35) have 4 cells while 43% and 38.5% had 6 cells, respectively. The trend in *spt-2* is similar (56% of observed roots had 6 or more cells in the QC (n = 35)); however, L. *er* tends to have more than 4 cells in its QC as well (40% of observed roots had 6 or greater cells (n = 35)).

**Figure 4:**
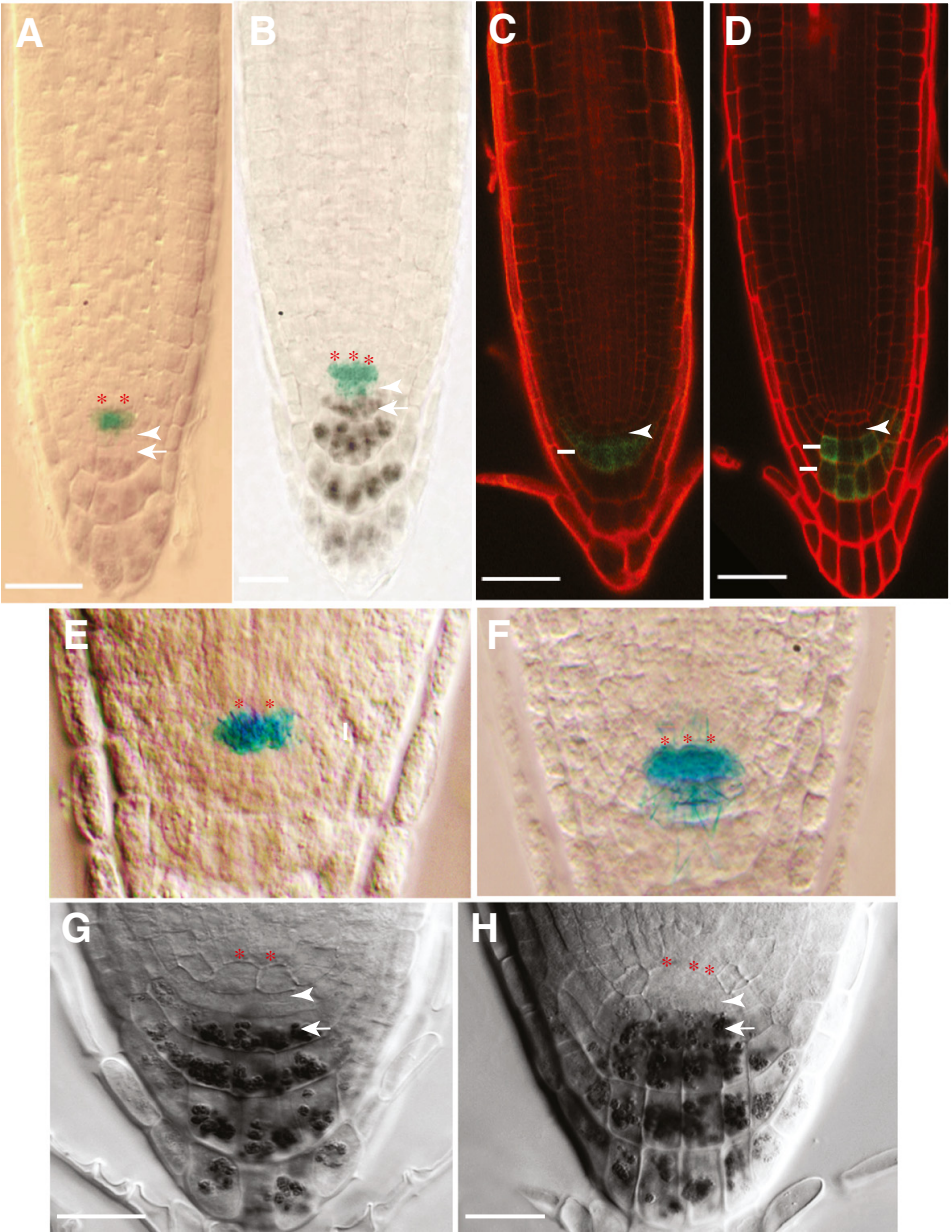
**QC size but not patterning is altered in *spt-11.*** Micrographs of 5 DAG roots. (**A**, **C**, **E**) Col-0. (**B**, **D**, **F**) *spt-11*. (**A**, **B**) Expression of *QC25::GUS* (blue) in the QC and starch in the differentiated columella. Red asterisks indicate QC cells, arrowheads indicate root cap initials and arrows indicate first row of columella cells. (**C**, **D**) Expression of the columella marker *QC1630::GFP* in propidium iodine-stained roots. Arrowheads indicate root cap initial cells and lines indicate stained columella cell layers. (**E**, **F**) Close-ups of the roots shown in A and B to highlight expression of *QC25::GUS.* Red asterisks indicate QC cells. (**G**, **H**) Micrographs of 5 DAG roots stained for starch. (**G**) L. *er*. (**H**) *spt-2*. Red asterisks appear above cells in the QC. Arrow indicates starch staining in the root cap. Arrowheads indicate columella and epidermal/lateral root cap cells. Scale bars indicate 100 μm.

Postembryonically, stem cell organization was studied by double staining of root tips for expression of GUS driven by a QC-specific promoter trap, *QC25*[28], and starch staining of root cap cells. *spt-11* has a single columella stem cell layer as in wild type and the root cap has differentiated normally (Figure 4B, F). However, *spt-11* root tips have more cells in the QC when compared to wild type (Figure 4B, F). In wild type, 81% of the observed seedlings (n = 32) had four cells marked by *QC25* expression while the other 19% had 6 cells in the QC. In *spt-11* seedlings only 30% of the observed seedlings (n = 51) have 4 cells displaying *QC25* expression while 38% had 6 cells, 30% had 8 cells and 2% had 10 cells in the QC. The increased number of cells in the QC was reflected in a broader QC (with more than two cell across) as well as QCs with more than one layer of cells. A low level of GUS staining driven by *QC25* (when compared to QC specific GUS staining) was also observed in the columella stem cell layer of *spt-11* root tips (Figure 4F). Consistent with the *QC25* results, the columella specific enhancer trap *Q1630::GFP* also reveals the presence of a single columella stem cell layer in *spt-11* (Figure 4D). Starch staining was also used to examine RAM organization in *spt-2* and L. *er* (Figure 4G, H). Consistent with the *spt-11* results, *spt-2* roots have a single layer of columella stem cells but extra QC cells (Figure 4H). This suggests that *SPT* function helps control the number of cells in the QC both in the embryonic and post embryonic stages, but not distal meristem organization.

### Loss of *SPT* function leads to a broader auxin maximum at the root tip but does not disrupt root patterning

**Figure 5:**
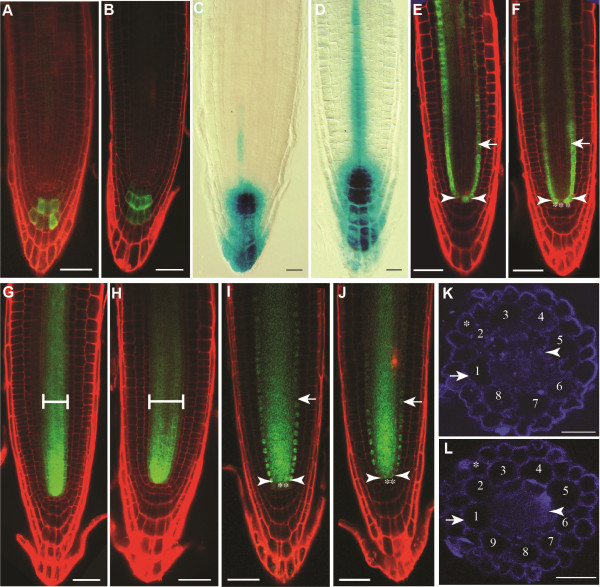
**Loss of *SPT *function alters auxin accumulation but does not disrupt root patterning.** (**A, B**) Confocal micrographs of 5 DAG roots stained with propidium iodide expressing *PIN4p::PIN4-GFP*. (**A**) Col-0. (**B**) *spt-11*. (**C, D**) Micrographs of 5 DAG roots expressing *DR5::GUS*. (**C**) Col-0. (**D**) *spt-11*. (**E-J**) Confocal micrographs of 5 DAG roots stained with propidium iodide. (**E, F**) *pSCRp::GFP* expression. Arrows indicate endodermal cells, asterisks indicate QC cells and arrowheads indicate vascular initials. (**E**) Col-0. (**F**) *spt-11*. (**G, H**) *pSHR::GFP* expression. The bracketed region is the stele. (**G**) Col-0. (**H**) *spt-11*. (**I-J**) *pSHR::SHR-GFP* expression. Arrows indicate endodermal cells, asterisks indicate QC cells and arrowheads indicate vascular initials. (**I**) Col-0. (**J**) *spt-11*. (**K, L**) Fluorescent brightener stained free hand cross sections of 7 DAG primary roots. Asterisks indicate the epidermal cell layer. Arrows indicate the cortical cell layer. Arrowheads indicate the endodermal cell layer. Cortical cells are numbered around the diameter. (**K**) Col-0. (**L**) *spt-11*. Scale bars in A-B and D-J indicate 100 μm. Scale bars in B-C and K-L indicate 50 μm.

Auxin accumulates at the distal root primordia and is required for QC and stem cell specification [29]. Previously it has been shown that *spt-2* gynoecial phenotypes were partially rescued by the application of the polar auxin transport inhibitor N-1-naphthylphthalmic acid (NPA), suggesting a role for *SPT* in control of auxin transport [30], presumably through its control of expression of *WAG2* and *PID*[19]. To test whether the auxin distribution or transport are altered in *spt* mutants, we looked at the expression of the auxin efflux carrier *PIN4*[31] and the auxin responsive reporter *DR5::GUS,* which is used to visualize auxin response maxima [32]. The *PIN4p::PIN4-GFP* and *DR5::GUS* transgenes were introduced into the *spt-11* background by crossing. PIN4-GFP accumulated in a nonpolar manner in the region of the QC and surrounding cells (Figure 5A). This expression pattern is similar to that documented by Friml et al. [31]; however, significant differences in the pattern of PIN4-GFP accumulation in wild type have been reported [33-35]. However, in our hands the expression is most evident in this region of the root [36]. *spt-11* has a broader expression domain of both *PIN4* (Figure 5B) and *DR5::GUS* (Figure 5D) when compared to wild type, suggesting a broader auxin maximum or an increase in auxin sensitivity, which might contribute to a larger QC and RAM in these plants.

In order to determine if loss of *SPT* function affects auxin sensitivity and/or transport, we tested the sensitivity of *spt-11* seedling root growth to the synthetic auxin 1-naphthaleneacetic acid (NAA) and the auxin transport inhibitor NPA. The inhibition of *spt-11* root growth by NAA was not significantly different from Col-0 (Table 2), suggesting that the sensitivity to exogenous auxin is not altered in this genetic background. In contrast, *spt-11* root growth was significantly more inhibited by NPA than wild type, although the difference became negligible at higher levels of NPA application (Table 3). The stronger *DR5::GUS* maxima in the *spt-11* background, combined with the increased sensitivity of *spt-11* root growth to NPA application supports the hypothesis that SPT may act to regulate polar auxin transport, directly or indirectly.

**Table 2:** **Response to exogenous NAA is not changed by loss of *SPT *** **function**

**Genotype**	**Concentration of NAA (nM)**	**Root length (mm)**	**% of mock**
Col-0			
	0	24.7 ± 6.7	NA^a^
	1	17.1 ± 4.2	69%
	20	4.6 ± 1.1	19%
	40	3.4 ± 1.0	16%
	60	3.1 ± 0.7	13%
	80	2.7 ± 0.7	11%
	100	2.2 ± 0.6	9%
*spt-11*			
	0	26.2 ± 3.7	NA
	1	17.7 ± 3.0	68%
	20	5.2 ± 1.7	20%
	40	4.8 ± 1.6	18%
	60	3.5 ± 0.9	13%
	80	3.2 ± 1.0	12%
	100	2.7 ± 1.4	11%

Values are means ± standard deviation. ^a^NA, not applicable.

**Table 3:** **Loss of *SPT *** **function confers increased sensitivity to NPA**

**Genotype**	**Concentration of NPA (μM)**	**Root length (mm)**	**% of mock**
Col-0			
	0	17.5 ± 3.3	NA^a^
	0.1	9.5 ± 1.5	55%
	0.5	4.3 ± 1.0	25%
	1	3.6 ± 0.7	21%
	2μM	2.2 ± 0.6	13%
*spt-11*			
	0	23.4 ± 2.9^b^	NA
	0.1	8.0 ± 1.0^b^	34%
	0.5	4.6 ± 1.0^b^	20%
	1	3.8 ± 0.4^b^	16%
	2	2.7 ± 0.5	12%

Values are means ± standard deviation. ^a^NA, not applicable. ^b^Values significantly different from wild type at P < 0.05.

Root stem cell niche specification and radial patterning are regulated in part by two transcription factors, SHR and SCR [8-10,37]. Since *spt-11* has more cells in the QC (Figures 3, 4), we looked at the expression of *SCR* and *SHR* to see if they are disrupted. *pSCR::GFP* is expressed in the endodermal layer, endodermal/cortical initials and QC (Figure 5E; [38]) while *pSHR::GFP* is expressed in the stelar cells (Figure 5G; [10]) and *pSHR::SHR-GFP* is expressed in the stelar tissue, endodermal cell layer, endodermal/cortical initials and QC (Figure 5I; [11]). No differences in the expression domains of these genes were seen in *spt-11* when compared to wild type seedlings (Figure 5F, H, J), indicating that *SPT* does not regulate stem cell niche positioning or radial patterning.

*SPT* expression has been detected in vascular tissues [39]. In order to check if *SPT* has any role in the development of vascular elements, we looked at the expression of two vascular markers. Enhancer trap *J0121::GFP* is specifically expressed in the xylem-associated pericycle cells (Additional file 3A; [40]) and the marker *CoYMV*::*GFP* is specifically expressed in the phloem companion cells (Additional file 3C; [41]). The expression of these markers in *spt-11* is similar to wild type expression patterns (Additional file 3B, D), suggesting that loss of *spt* does not disrupt differentiation of the vasculature. We also looked at whether the radial organization of the root is affected in *spt-11*. Col-0 roots have 8 cells in the cortical cell layer (Figure 5K; n = 15 seedlings; >20 sections/seedling) as previously reported [1], whereas *spt-11* roots have 9 cells in the cortical cell layer (Figure 5L; n = 15 seedlings; >20 sections/seedling), likely reflecting the increased cell division in the root meristem. However, the overall organization of the root is unaffected.

### *SPT* acts in parallel to GA in the root

**Figure 6:**
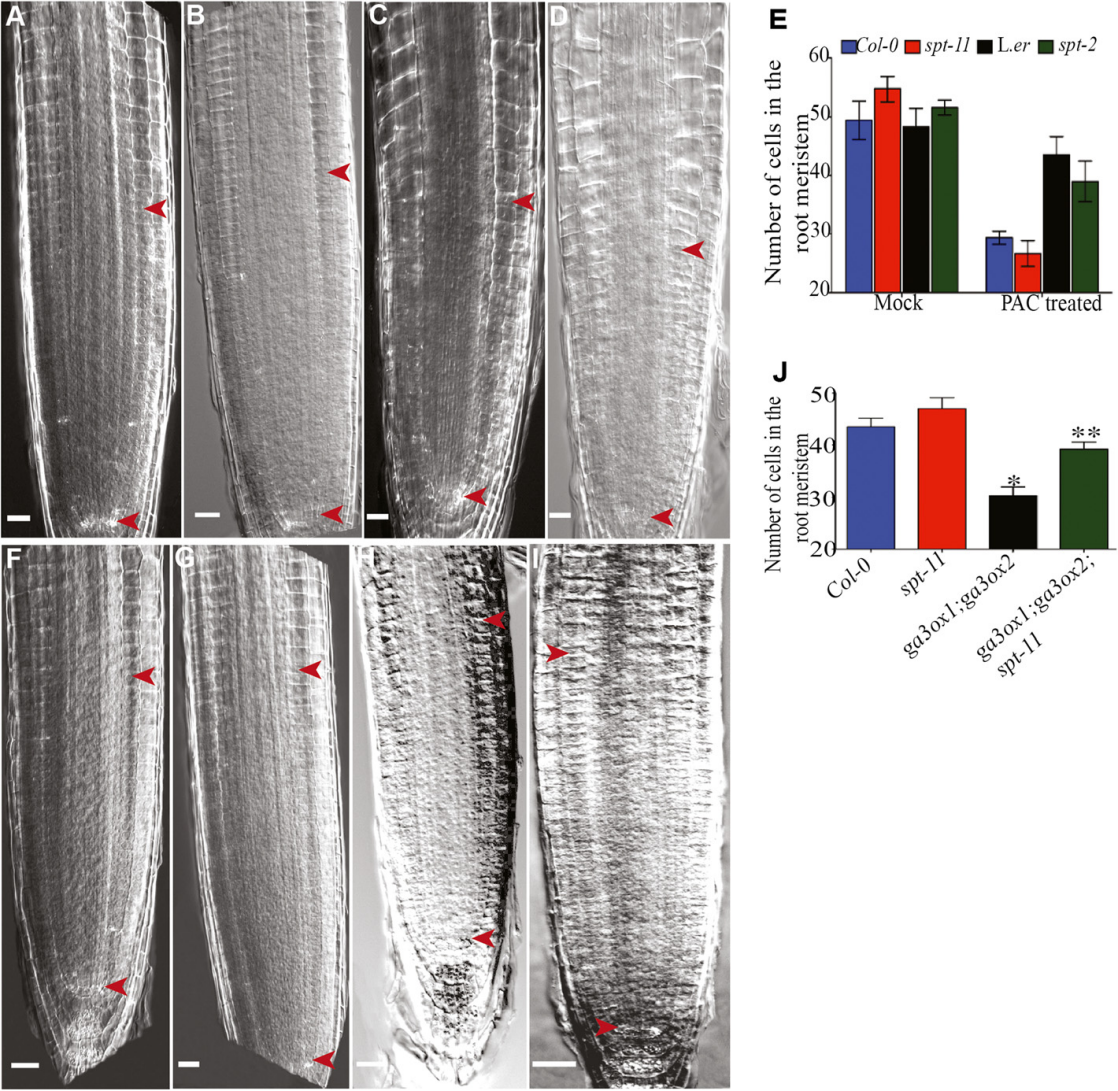
***SPT *acts additively with GA in the root.** (**A-D**) Micrographs of 8 DAG roots. Arrowheads indicate the zone of cell proliferation. (**A, B**) Mock treated. (**C, D**) PAC treated. (**A, C**) Col-0. (**B, D**) *spt-11*. (**E**) RAM cell number of mock and PAC treated seedlings. Error bars indicate standard error. The values are means of three independent experiments (n = 15/replicate). (**F-I**) Micrographs of representative 7 DAG seedlings. Arrowheads indicate the zone of cell proliferation. (**F**) Col-0. (**G**) *spt-11*. (**H**) *ga3ox1-2; ga3ox2-1*. (**I**) *ga3ox1-2; ga3ox2-1; spt-11*. (**J**) RAM cell number (n = 15). Asterisks and double asterisks indicate values that are significantly different from wild type and *ga3ox1-2; ga3ox2-1*, respectively. Scale bars indicate 100 μm.

GA is known to play a role in regulating RAM size [42,43] and *SPT* has been implicated in regulation of GA biosynthesis and in GA signalling [20,23] as well as shown to act in parallel to the GA pathway [23]. The relationship between GA and SPT in the root is unknown, however. In order to determine whether *spt* mutants are responsive to reduced levels of GA, we exposed seedlings to the GA biosynthesis inhibitor paclobutrozol (PAC) and examined the effect on meristem size. As expected, meristem size was reduced in wild type upon exposure to PAC (Figure 6B, E). PAC also reduced meristem size in *spt* mutants (Figure 6D, E). Interestingly, the response to PAC in L. *er* roots is not very robust and *spt-2* responds more to this inhibitor than L. *er* (Figure 6E). It has previously been shown that L. *er* is saturated for GA response in the cotyledons, although it displayed a robust response to PAC in those organs [23]. L. *er* may have increased levels of GA in roots that buffer its response to GA biosynthetic inhibitors.

To further investigate the relationship between *SPT*-mediated cell proliferation and GA, we crossed *spt-11* and *spt-2* mutants to GA biosynthesis mutants in the appropriate genetic backgrounds, *ga3ox1-2*; *ga3ox2-1*[44] and *ga1-3*[45], respectively. Root meristem size was examined in 7-day-old seedlings of Col-0, *spt-11, ga3ox1-2*; *ga3ox2-1* and the triple mutant *ga3ox1-2*; *ga3ox2-1*; *spt-11*. The double mutant of *ga3ox1-2*; *ga3ox2-1* has a short RAM, as previously reported (Figure 6H, J; [42]) while *spt-11* has the longest RAM among all the genotypes analyzed (Figure 6G, J). The triple mutant *ga3ox1-2*; *ga3ox2-1*; *spt-11* has a significantly bigger RAM than that of double mutant *ga3ox1-2*; *ga3ox2-1*, but a smaller RAM than *spt-11* (Figure 6I, J), an additive phenotype. This triple mutant and the *ga1-3; spt-2* double mutant were analyzed for other developmental differences. *ga3ox1-2*; *ga3ox2-1*; *spt-11* plants as well as *ga1-3; spt-2* plants are of intermediate height compared to their parents (Additional files 4 and 5). Triple mutants flowered significantly earlier than the *ga3ox1-2; ga3ox2-1* plants, have significantly higher number of flowers and internodes, significantly longer internodes and are significantly taller (Additional file 5), suggesting that the loss of *SPT* function can partially compensate for lower GA levels in the plant. However, the triple mutant plants are neither equivalent to *spt-11* nor wild type plants. This additive phenotype suggests that *SPT* and GA act in parallel pathways. The fruit phenotype of *spt-11* is still retained in *ga3ox1-2*; *ga3ox2-1*; *spt-11* plants (data not shown), suggesting either that GA is not functioning in apical carpel development or that *SPT* acts downstream of GA in this context.

**Figure 7:**
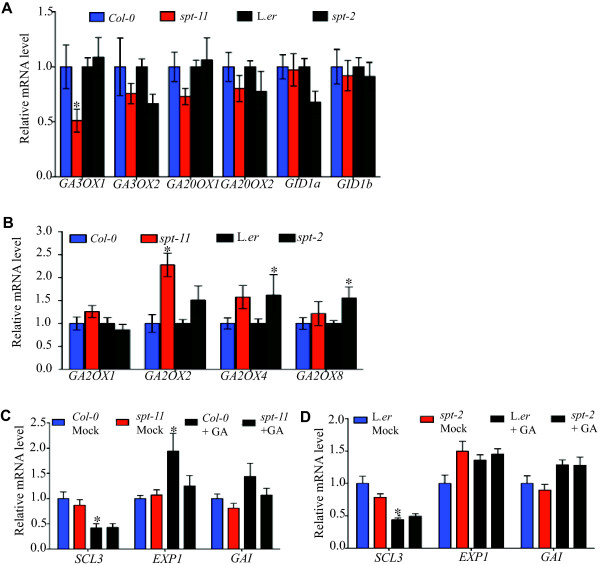
**GA-responsive gene expression in the *spt *background.** 7-day-old seedlings were used for gene expression experiments. For analysis of GA-responsive genes, seedlings were treated with 50μM GA or mock solution for three hours. (**A**) Expression of GA biosynthesis genes and GA receptor-encoding genes. (**B**) Expression of GA catabolism genes. (**C, D**) Expression of GA-responsive genes. Error bars indicate standard error of the means of expression.

Endogenous GA levels are regulated by both GA biosynthesis and catabolism/deactivation. GA20-oxidases and GA3-oxidases are each encoded by multi-gene families and catalyze the final steps in GA biosynthesis pathway [46]. These genes are expressed at higher levels in GA-deficient backgrounds and their expression decreases after application of bioactive GAs [47-51]. Endogenous GA levels are regulated by metabolic deactivation and the GA2-oxidases are the best characterized enzymes shown to catalyze such a reaction [51,52]. In contrast to the expression of GA biosynthesis genes, *GA2-OXIDASE* levels go up upon GA application [52-54]. The genes encoding the soluble GA receptors, *GID1a* and *GID1b*, whose expression goes down upon GA treatment [52,55], are another marker of GA levels in Arabidopsis. We examined expression of a subset of these genes in 7-day-old seedlings of *spt* mutants and their wild types by qPCR analysis. Among the GA biosynthesis genes analyzed, expression of only one (*GA3ox1)* changed significantly from wild type and only in *spt-11*, where it was reduced (Figure 7A). Among the GA deactivation pathway genes checked, transcript levels of *GA2ox2* was significantly higher in *spt-11* and *GA2ox4* and *GAox8* transcript levels were higher in *spt-2* (Figure 7B). The expression of the GA receptors did not significantly change in the mutants (Figure 7A). Thus, there was not a consistent change in gene expression that would indicate that GA levels or signalling are altered in the *spt* background.

Since the above expression results were inconclusive, we examined the expression of several downstream GA-responsive genes: *SCARECROW-LIKE 3 (SCL3)*, which is downregulated in response to GA, and *EXPANSIN1 (EXP1)* and *GIBBERELLIC ACID INSENSITIVE* (*GAI)*, both of which are increased by GA [56]. Consistent with previous reports, exogenous GA does not significantly increase GA-induced gene expression in the L. *er* background (Figure 7D; [23]). In the absence of GA application, there was no significant difference in expression of *SCL3*, *EXP1* or *GAI* between *spt-11* or *spt-2* and wild type (Figure 7C, D). Upon GA application, the significant increase in *EXP1* expression seen in wild type is not observed in *spt-11* (Figure 7C), perhaps suggesting that *SPT* contributes to regulation of this gene. However, similar to the situation seen with the GA biosynthetic and catabolic genes, no consistent pattern of GA responsive gene expression was seen.

## Discussion

Previous studies have shown that *SPT* functions in diverse organs of the aerial portion of Arabidopsis, including the cotyledon [23], the leaf [21], the gynoecium [16,17,57], the fruit [18,19] and in germinating seeds [20]. In this study we have shown that *SPT* also functions in the root, where it acts to restrict RAM size and root length. Loss of function *spt* mutants have a larger zone of cell division (Figure 1), which contains more dividing cells than wild type (Additional file 2); this leads to a higher growth rate in the roots and longer primary roots. In adult plants, the inflorescence stem is significantly longer than that of wild type and produces more flowers (Table 1). It has previously been shown that *spt-11* plants have larger leaf areas due to increased cell number [21] and cotyledon size due to increased cell expansion [23]. Taken together, these data suggest that *SPT* acts to restrict cell proliferation and expansion in a number of organs in Arabidopsis.

QC size was increased in both *spt-2* and *spt-11* roots, as assayed by morphology and molecular markers. In the wild type background, the QC consists of on average four cells that are mitotically less active than the surrounding initial cells [1]. However, *spt* mutants have an increased number of cells in their QCs, often being three or four cells across instead of two and sometimes having two layers of QC cells (Figures 3, 4). The increase in size of the QC is evident in the embryo, starting at approximately torpedo stage (Figure 3). *spt-11* embryos can have up to 6 cells in their QC and the size increase continues during root development, as roots with up to 10 QC cells were observed. The increase in the size of the QC and the root division zone in roots of *spt* mutants is similar to the increase in the size of the meristematic region of leaves in *spt* mutants [21], suggesting that the molecular pathway in which *SPT* functions may be similar in these two organs.

*SPT* expression is correlated with areas of high auxin content [16,24], suggesting a relationship between *SPT* and auxin. The *SPT* promoter also contains several auxin response elements (AREs), suggesting that auxin response factors (ARFs) may directly regulate its expression. However, it has previously been shown that mutating these elements does not change expression of a *SPT* reporter [24]. The increase in size of the RAM seen in *spt* mutants is correlated with a broader zone of expression of the auxin efflux carrier *PIN4* and a stronger auxin maximum, as visualized by *DR5::GUS* expression (Figure 5). This may result from changes in auxin transport, as seen in developing carpels of *spt* mutants [19,30], which is supported by the increased sensitivity to NPA shown by *spt-11* roots (Table 3). *spt* carpel defects can be rescued by application of the auxin transport inhibitor NPA [30,57], suggesting that *SPT* activity may impact auxin transport, which is consistent with its regulation of protein kinases that regulate the PIN efflux carriers [19]. However, in the root, *spt-11* mutants are hypersensitive to NPA application (Table 3) while in the carpel its application ameliorates the developmental defects caused by loss of *SPT*. A possible explanation for this could be differences between auxin transport in the carpel and the root tips. Carpel development depends on an auxin gradient along the apical-basal axis of the gynoecium, with the highest level of auxin present in the apex. Root development and growth, however, depends on both on an apical-basal auxin gradient with its greatest concentration in the region of the QC (generated by polar transport through protophloem cells) and redirection of that auxin flow laterally in the root cap where it subsequently flows back toward the shoot (through lateral root cap and epidermal cells) [58]. Disruption of any of these auxin transport pathways impacts root growth and development [34,59]. Thus, NPA application on the root tip likely causes more complex changes in auxin flow and accumulation compared to its effect in the carpel. SPT may regulate not only apical-basal auxin transport but also the auxin redirection pathway as well.

Our data, similar to that reported by other groups working on shoot organs [23], suggests that SPT functions in parallel to GA to regulate RAM size and root length. It has long been known that there is crosstalk between auxin and GA and between auxin transport and GA. Recently it has been shown that GA-deficient plants accumulate fewer PIN auxin transport proteins, although PIN4 accumulation was not evaluated, and that this correlates with less auxin transport [60]. Therefore, it is possible that changes in GA content or signalling in *spt* mutants might lead to the changes in auxin accumulation at the root tip we observed. Clearly more work is necessary to determine the relationship(s) between GA, auxin and *SPT*.

As mentioned above, *SPT* has functions in germination, cotyledon expansion, leaf size and gynoecium development. Our work extends the functions of this gene into the root, where it acts to regulate cell proliferation in the meristematic zone without impacting overall root organization or differentiation in the mature area of the root. This is similar to the role of *SPT* in leaf growth control, where it appears to act by restricting the size of the basal meristematic zone of the leaf without altering leaf morphology or cell types [21]. This is in contrast to the effect of loss of *SPT* in the flower, where less cell proliferation takes place in the gynoecium, resulting in a shorter pistil with defects in stigma, style and transmitting tract tissues [17]. However, since *SPT* may act through regulation of auxin transport in both the carpel and root, the varying impacts on cell proliferation in these organs may be due to differences in auxin response.

*SPT* encodes a bHLH protein [16] that has been shown to act as a transcriptional activator [61]. bHLH proteins act in dimers or larger order protein complexes. SPT belongs to a subclade of bHLH factors (Group VII of [62]/subfamily 15 of [63]). This group has fourteen members of which the *ALC* gene, partially redundant with *SPT*[18,64], is most closely related. These proteins can heterodimerize with each other [18]; however, *ALC* is not highly expressed in the root (Genevestigator; [65,66]). In addition, the PIF/PIL bHLH proteins fall in this clade, which interact with phytochromes and contain a PHYB-binding domain not found in either SPT or ALC [63,67]. *SPT* has been shown to interact genetically with *PIL5* during seed germination [20] and PIL5 is known to regulate GA responsiveness [68]. SPT also interacts with *PIF6* during pistil development [57], suggesting it may be able to act with the products of these genes to regulate gene expression. However, root expression of these genes is low (Genevestigator; [65,66]). SPT also heterodimerizes with members of the HECATE family of bHLH transcription factors [69]. Loss of these genes causes carpel defects similar to those of *spt* mutants and they are expressed in an overlapping pattern with *SPT* in the carpel, but are not expressed in the root. Additionally, SPT interacts with IND in the carpel and fruit where it may bind DNA cooperatively with that protein [19]; it is unknown if this gene is expressed in roots.

While no bHLH proteins have been shown to interact with SPT in the root to date and its known interactors are not known to be expressed in this organ, at least three genes encoding bHLH transcription factors are active in root growth control. *LONESOME HIGHWAY (LHW)* regulates the size of the stem cell pool that gives rise to the cells of the root vascular cylinder [40], while UPBEAT1 (UPB1) regulates the expression of peroxidases to modulate the balance of reactive oxygen species between the zone of cell division and the elongation zone, regulating the onset of differentiation [70]. Expression of both of these factors partially overlaps with that of *SPT*. In addition, MYC2 has been shown to be necessary for the jasmonate-mediated repression of root growth by directly repressing expression of *PLT1* and *PLT2*[71]. Examination of binding partners of SPT in the root and identification of target genes in this organ will provide great insight into the molecular pathway or pathways in which *SPT* acts.

## Conclusions

*SPT* has previously been shown to regulate growth in several above ground organs of Arabidopsis. *SPT* also regulates proliferation in the root, controlling the size of the RAM and the number of cells in the QC. However, the organization of the root and differentiation of root cell types is not altered, although extra cells are made. *SPT* regulates growth in parallel to GA and by modifying the accumulation of auxin in the region of the QC, likely via regulation of auxin transport.

## Methods

### Plant materials and growth conditions

The *spt-2* allele is in the L*. er* background and has been previously described [17]. The *spt-11* allele, a T-DNA insertion line in the Col-0 background, has been previously described [21] and is from the WISCDSLOX collection [72]. Seeds of other mutants used in the study have also been described: *ga1-3*[45] and *ga3ox1-3*; *ga3ox2-1*[44]. The double mutant *ga1-3*; *spt-2* was generated by crossing homozygous *ga1-3* and homozygous *spt-2* plants and allowing the F1 to self-fertilize. The triple mutant *ga3ox1-3*; *ga3ox2-1*; *spt-11* was generated by crossing homozygous *ga3ox1-3*; *ga3ox2-1* plants and homozygous *spt-11* plants and allowing the F1 to self-fertilize. Double and triple mutants were identified by PCR genotyping of the segregating F2 population. To generate *spt-11* mutants containing marker lines, homozygous *spt-11* plants were crossed to the lines (Additional file 6) and the F1 allowed to self-fertilize. Mutants were identified by PCR genotyping of the F3 after growth on antibiotic containing media to select for the transgene.

*Arabidopsis thaliana* seeds were cold treated for 3 days at 4°C and were germinated and grown on Fafard 2 mix soil (Fafard) under long-day (16 hours, 80 μmol m^-2^ s^-1^) irradiance, either in controlled growth chambers (Enconair Ecological Chambers Inc., Manitoba, Canada) or growth rooms with subirrigation at 22°C with 60% relative humidity.

Seeds used in all the assays done on seedlings were sterilized as previously described [73], placed on either on Murashige & Skoog (MS) media (Research Products International Corporation, Mt. Prospect, IL) with 1% plant agar in Petri plates or on half GM plates (half concentration of MS salts, 1% sucrose, 0.8% Plant agar, pH 5.7). The plates were incubated in the dark at 4°C for 5 days. The plates were then moved to a CU-36L growth chamber (Percival Scientific Inc., Perry, IA), placed vertically and grown under long day conditions as above unless noted.

### PCR genotyping

Mutants were identified by PCR genotyping of genomic DNA. Genomic DNA was extracted from inflorescences and leaves as described previously [73]. Primer sequences are shown in Additional file 7 and combinations used for genotyping various mutants are listed in Additional file 8.

### Phenotypic analysis

In order to analyze various developmental phenotypes of mutants and wild type, plants were grown under long day conditions. Seeds of various genotypes were sown in square cells or pots in a randomized block design. After seeds were germinated, all seedlings except one per cell or pot were weeded out.

Several aerial phenotypes were analyzed as previously described [73]. Leaf number at flowering was defined as the number of leaves when the first flower opened. In order to analyze root phenotypes, plants were grown on vertically oriented plates. For all the measurements done on seedlings in this study, the first day of incubation in the chamber was counted as day zero. Seedlings were collected for analysis at different Days After Germination (DAG) starting at 3 DAG till 11 DAG. To visualize roots using microscopy, seedlings were fixed overnight in ethanol and acetic acid (9:1). Roots were cleared in chloral hydrate (80 grams of chloral hydrate, 20 ml of water and 10 ml of glycerol) on microscope slides for 20 minutes for microscopic analysis with Differential Interference Contrast (DIC) optics on a Nikon Eclipse 90i. Pictures were taken using the attached Nikon camera and analyzed with NIS elements Advanced Research software version 3.0. Fifteen seedlings per genotype were used for root meristem size measurement. Root meristem size was measured as the number of cells in the cortical cell layer between the QC and the first elongating cell as described [25] and the results were depicted in graphical format using Prism (http://www.graphpad.com/prism/; GraphPad Software, La Jolla, CA). Results were analyzed statistically using a two-sample student t-test. The length of the meristematic zone was measured in micrometers from the QC to the first elongating cell in the root cortical cell layer as described [25]. Results were analyzed and displayed as above.

In order to determine the effect of PAC (PhytoTechnology Laboratories, Shawnee Mission, KS) on root growth, seedlings were transferred from half GM plates to half GM plates with 10μM PAC or half GM plates with methanol 4 days after germination. The seedlings were grown in the incubator for 96 hours before they were analyzed for root meristem size as above.

The average primary root length was determined using 30 seedlings of each genotype per replicate with 3 replications. Measurements were taken at 7, 10 and 13 DAG. Primary root growth rate was measured by drawing a line at the tip of primary root on the back of the plate every day, starting from 2 DAG. The distance between the two markings was measured with a ruler. The lengths between the two time points were used for obtaining the growth rate per hour (length in mm/24 hours).

To look at the cellular organization of roots, 5-day-old roots were hand-sectioned according to the protocol “Rapid preparations of transverse sections of plant roots” (http://www.mcdb.lsa.umich.edu/labs/schiefel/protocols.html). The cross-sections were cut perpendicular to the length of the root beginning at the root tip and moving towards the base of the root. The root sections were transferred to a Petri dish containing fluorescent brightener 28 (FB 28 Sigma-Aldrich, St. Louis, MO) dissolved in water. Sections were stained for 10 minutes and examined using UV epifluorescence microscopy using a Nikon Eclipse 90i Microscope. 15 seedlings of each genotype were examined and at least 20 sections per seedling were analyzed.

To visualize embryos for microscopy, seeds containing embryos at different stages of development were collected from developing fruits and processed as previously described [36]. Embryos were visualized with DIC optics on a Nikon Eclipse 90i. Pictures were taken using the attached Nikon camera and analyzed with NIS elements Advanced Research software version 3.0.

### Gene expression studies

To examine *SPT* expression in roots, roots of 7 DAG L. *er* seedlings grown on plates were collected and stored at -80. Total RNA from two biological replicates was extracted using the RNeasy Plant Mini Kit (Qiagen, Valencia, CA) according to instructions. During the RNA purification, on column DNase treatment was done using RNase-free DNase (Qiagen) and RNA was also treated with DNase again in solution. 500 ng of RNA was used as template and cDNA synthesis and PCR was done using the SuperScript III One-Step RT-PCR System (Invitrogen by Life Technologies) using *SPT* and *ACTIN*-specific primers (Additional file 7). For real-time PCR analysis, the Blue Print First Strand cDNA Synthesis Kit (Takara Bio Inc., Otsu, Shiga, Japan) was used with 1 μg of total RNA as template to generate cDNA. 0.5 μl of cDNA was used in real-time PCR (qPCR) reactions done using the iQ™ SYBR Green Supermix (BioRad) on a CFX96™ Real-Time PCR detection system (BioRad) at the PMGF. A reference gene, *At1g13320*, was used to normalize the qPCR data [74]. qPCR data was analyzed using CFX96 software and graphs were made using Prism. Primers for qPCR were designed using QuantPrime Q-PCR primer design tool (Additional files 7 and 9; http://www.quantprime.de; [75]).

For gene expression studies on GA response genes, seedlings were collected at 7 DAG and processed as above. Each genotype was represented by three biological replicates. For GA responsive gene expression, 7-day-old seedlings were treated with either 50 μM GA or mock in MS liquid media (1X MS salts, 1X Gamborg’s B5 Vitamins, 3% Sucrose with pH 5.7) for three hours in the incubator under constant light at 22°C. For quantifying GA biosynthesis and metabolism genes, three biological and two technical replicates were done. A reference gene, *At1g13320*, was used to normalize the qPCR data for GA biosynthesis and metabolism genes, while another reference gene, *At4g33380*, was used for normalizing the qPCR data for GA responsive genes [74]. Primers were designed as above. Results were analyzed statistically using a non-parametric Wilcoxon rank sum test for GA biosynthesis, catabolism and receptor genes and using a 2-way analysis of variance for GA responsive genes [76,77].

### ß-glucuronidase and starch staining

Expression of *QC25::GUS* was examined as previously described [36]. Photographs were taken using a Nikon Digital Sight DS-5M camera attached to a Nikon SMZ800 dissecting or on a Nikon Eclipse E200 compound microscope. GUS-stained 5-day-old seedlings were used for visualizing starch granule accumulation in the columella root cap cells. Staining for starch granules was done according to [78] in 1% lugol solution for 3 minutes, rinsed in water, cleared in chloral hydrate and photographed using Nomarski optics on a Nikon Eclipse 90i Microscope.

### Confocal microscopy

Confocal laser microscopy was used for looking at the expression of various cell specific markers tagged with Green Fluorescent Protein (GFP). The cell walls of various stages of embryos and roots were labelled with propidium iodide and were observed according to [36] with a Nikon D-Eclipse C1si Confocal.

### NAA and NPA assays

NAA and NPA assays were done as described in [79]. Briefly, seeds of Col-0 and *spt-11* were sown on MS media with varying concentrations of NAA (0, 1, 20, 40, 60, 80 and 100 nM) or NPA (0, 0.1, 0.5, 1 and 2 μM). Seeds were cold treated for two days and then grown vertically as described above. Root length of 8-day-old seedlings was measured as described above. The average data from three independent experiments are presented and at least 20 seedlings were analyzed per genotype per experiment.

## Abbreviations

RAM: Root apical meristem; QC: Quiescent center; PLT1: Plethora1; PLT2: Plethora2; SHR: Shortroot; SCR: Scarecrow; SPT: Spatula; ALC: Alcatraz; IND: Indehiscent; NPA: Naphthylphthalmic acid; NAA: 1-naphthaleneacetic acid; GA: Gibberellic acid; PAC: Paclobutrozol; L. er: Landsberg erecta; Col-0: Columbia-0; SCL3: Scarecrow-like 3; EXP1: Expansin1; GAI: Gibberellic acid insensitive.

## Competing interests

The authors declare that they have no competing interests.

## Authors’ contributions

SM and RSL conceived and designed the experiments. SM performed most of the experiments and analyzed the data. RSL performed the NAA and NPA experiments and RT-PCR on roots. SM and RSL wrote the paper. All authors read and approved the final manuscript.

## Supplementary Material

Additional file 1 ***SPT*is expressed in roots.** RT-PCR using total RNA isolated from L. *er* 7 DAG seedling roots (7 DAG). Two independent biological replicates are shown. *SPT* product is on the left and *ACTIN* product is on the right. (A) Sample 1. (B) Sample 2. Click here for file

Additional file 2 ***spt-11*RAMs contain more dividing cells.** Micrographs of 5 DAG root tips expressing the G2-M marker *CYCB1;1::GUS*. (A) Col-0. (B) *spt-11*. Click here for file

Additional file 3 **Vascular cell fate is not altered in*spt-11*mutants.** Micrographs of 5 DAG roots stained with propidium iodine. (A, B) Expression of the xylem-associated pericycle marker *J0121::GFP*. (C, D) Expression of the companion cell marker *CoYMV::GFP*. (A, C) Col-0. (B, D) *spt-11*. Click here for file

Additional file 4 ***SPT*acts additively with GA.** Photographs of adult plants. (A) Representative Col-0, *spt-11*, *ga3ox1-2; ga3ox2-1*, *ga3ox1-2; ga3ox2-1; spt-11/+* and *ga3ox1-2; ga3ox2-1; spt-11* plants. (B) Representative L*. er*, *spt-2*, *ga1-3*, *ga1-3; spt-2/+* and *ga1-3; spt-2* plants. Click here for file

Additional file 5 ***SPT*** **and GA act additively.** Click here for file

Additional file 6 Marker lines used in this study. Click here for file

Additional file 7 Primers used in this study. Click here for file

Additional file 8 Primer combinations used for genotyping. Click here for file

Additional file 9 Primer combinations used in qRT-PCR. Click here for file
