# Supplementary figures and images for: The bHLH transcription factor SPATULA regulates root growth by controlling the size of the root meristem
Source: BMC Plant Biol. 2013 Jan 2;13:1. doi: 10.1186/1471-2229-13-1 (PMC3583232; doi:10.1186/1471-2229-13-1)

**A.**

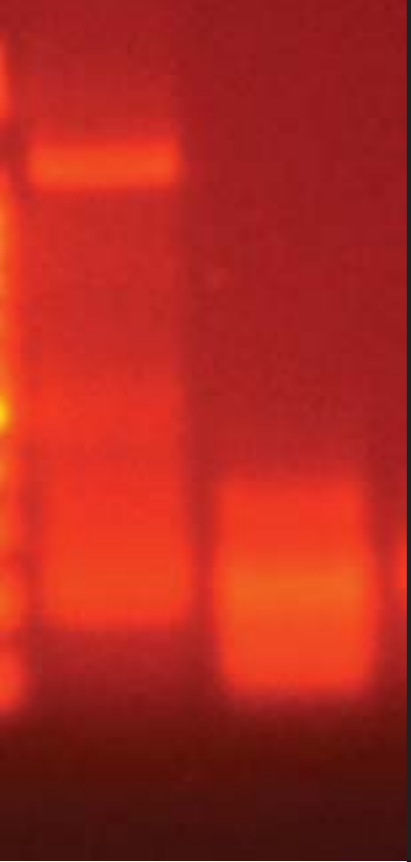

**B.**

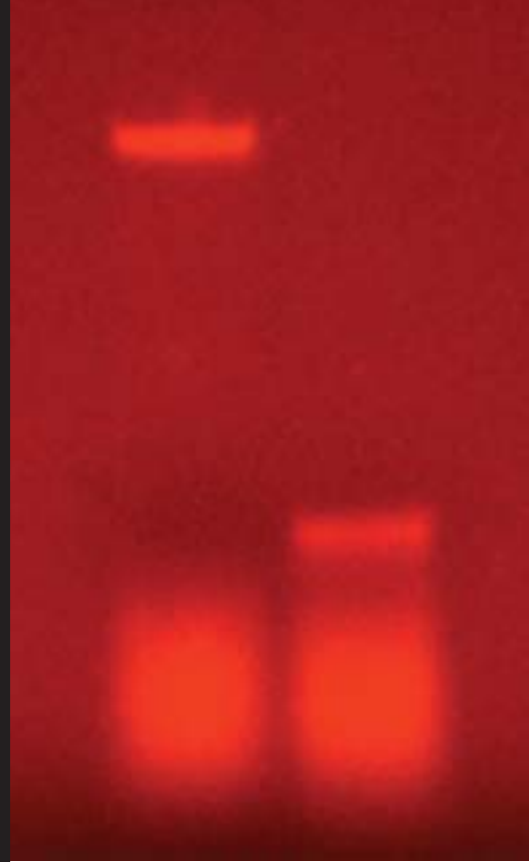

Supplement: Additional file 1 — SPTis expressed in roots. RT-PCR using total RNA isolated from L. er 7 DAG seedling roots (7 DAG). Two independent biological replicates are shown. SPT product is on the left and ACTIN product is on the right. (A) Sample 1. (B) Sample 2. [file 1471-2229-13-1-S1.pdf]

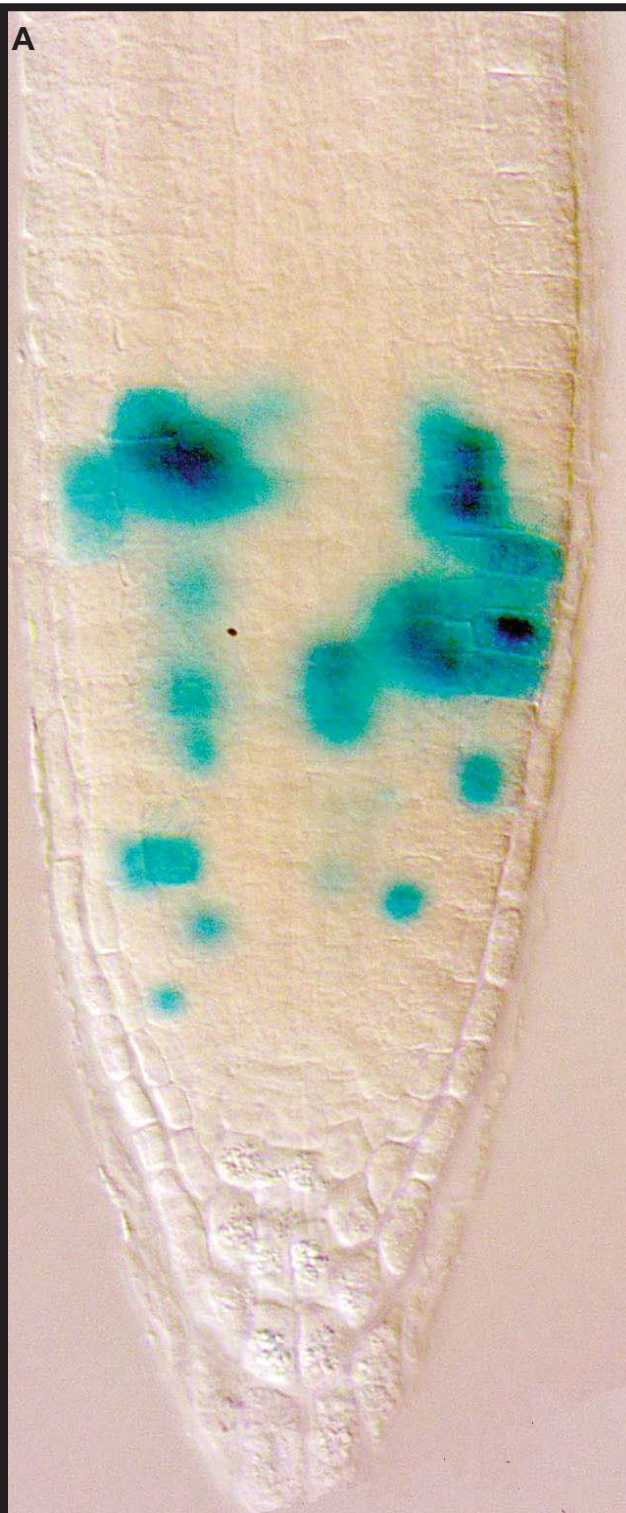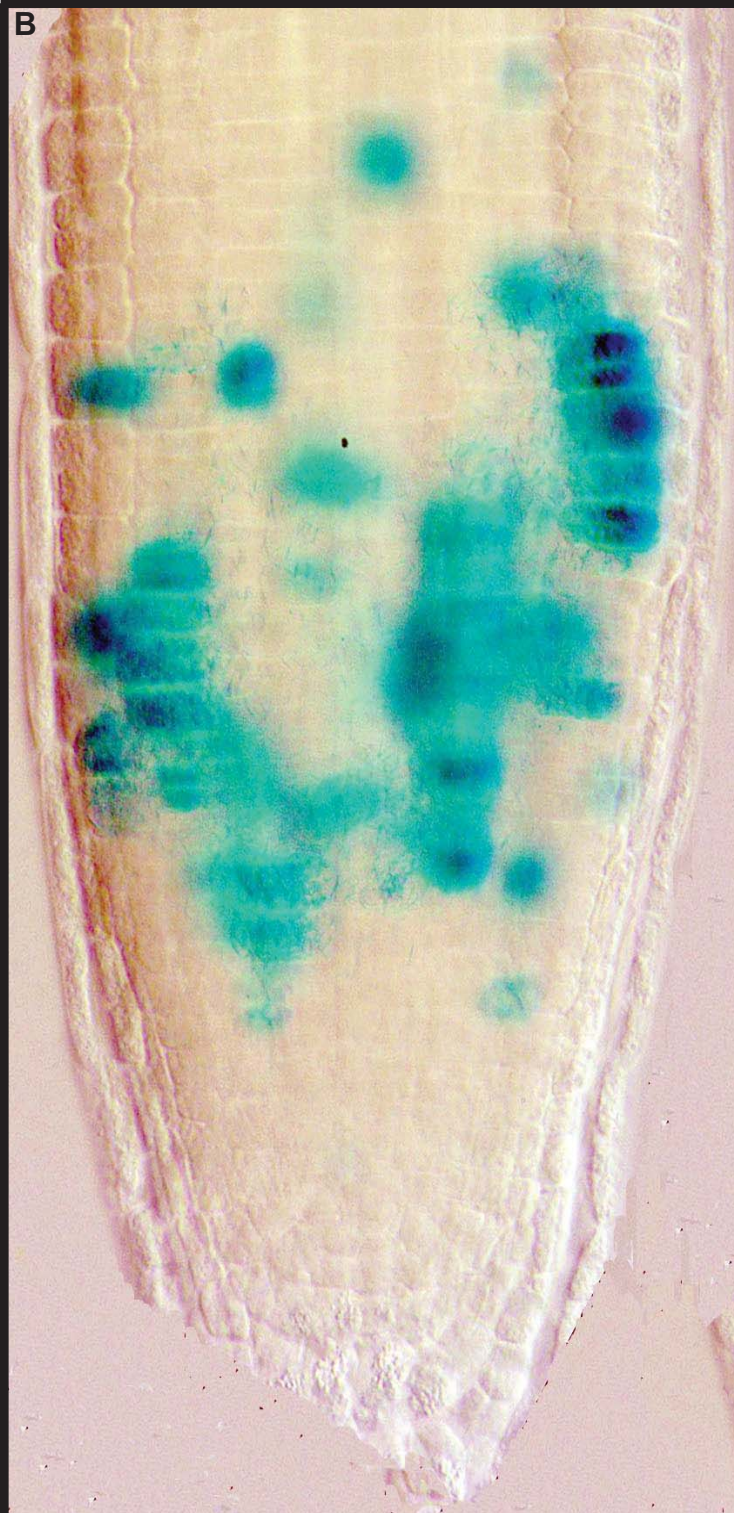

Supplement: Additional file 2 — spt-11RAMs contain more dividing cells. Micrographs of 5 DAG root tips expressing the G2-M marker CYCB1;1::GUS. (A) Col-0. (B) spt-11. [file 1471-2229-13-1-S2.pdf]

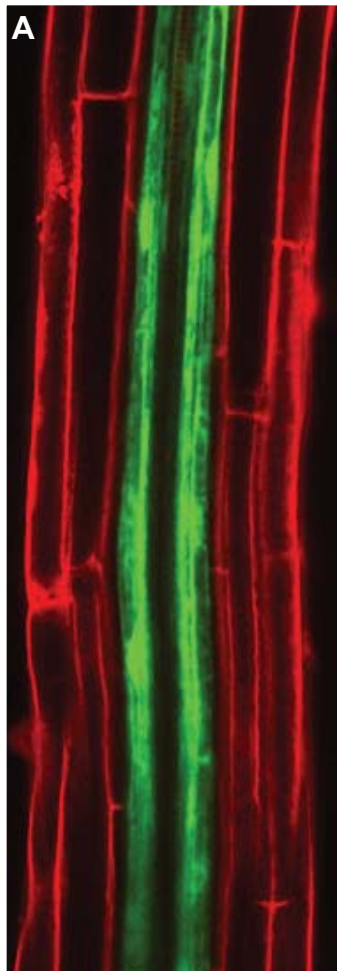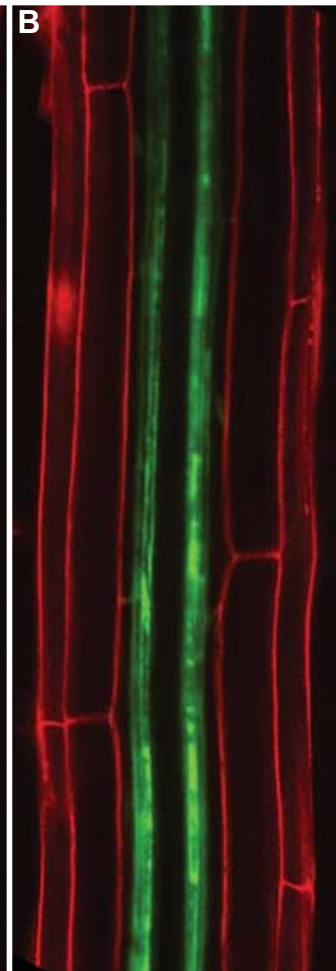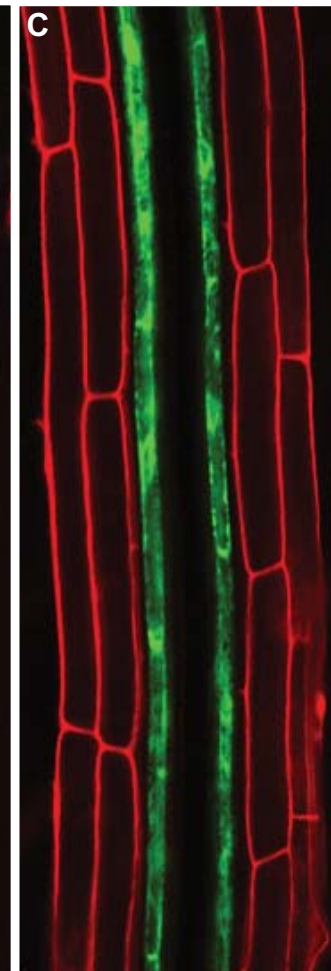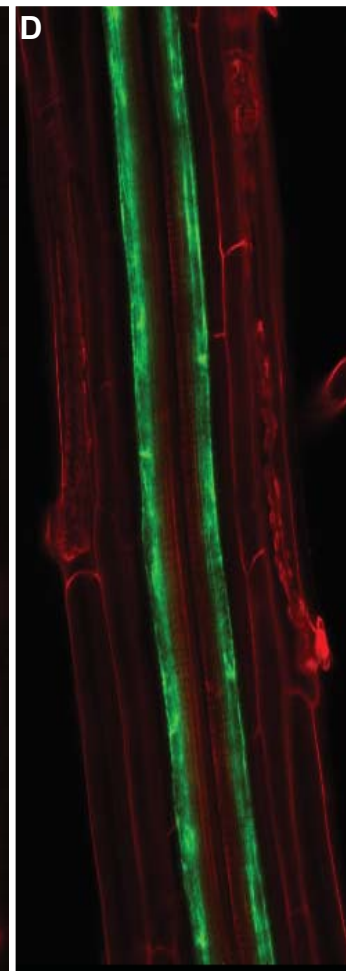

Supplement: Additional file 3 — Vascular cell fate is not altered inspt-11mutants. Micrographs of 5 DAG roots stained with propidium iodine. (A, B) Expression of the xylem-associated pericycle marker J0121::GFP. (C, D) Expression of the companion cell marker CoYMV::GFP. (A, C) Col-0. (B, D) spt-11. [file 1471-2229-13-1-S3.pdf]

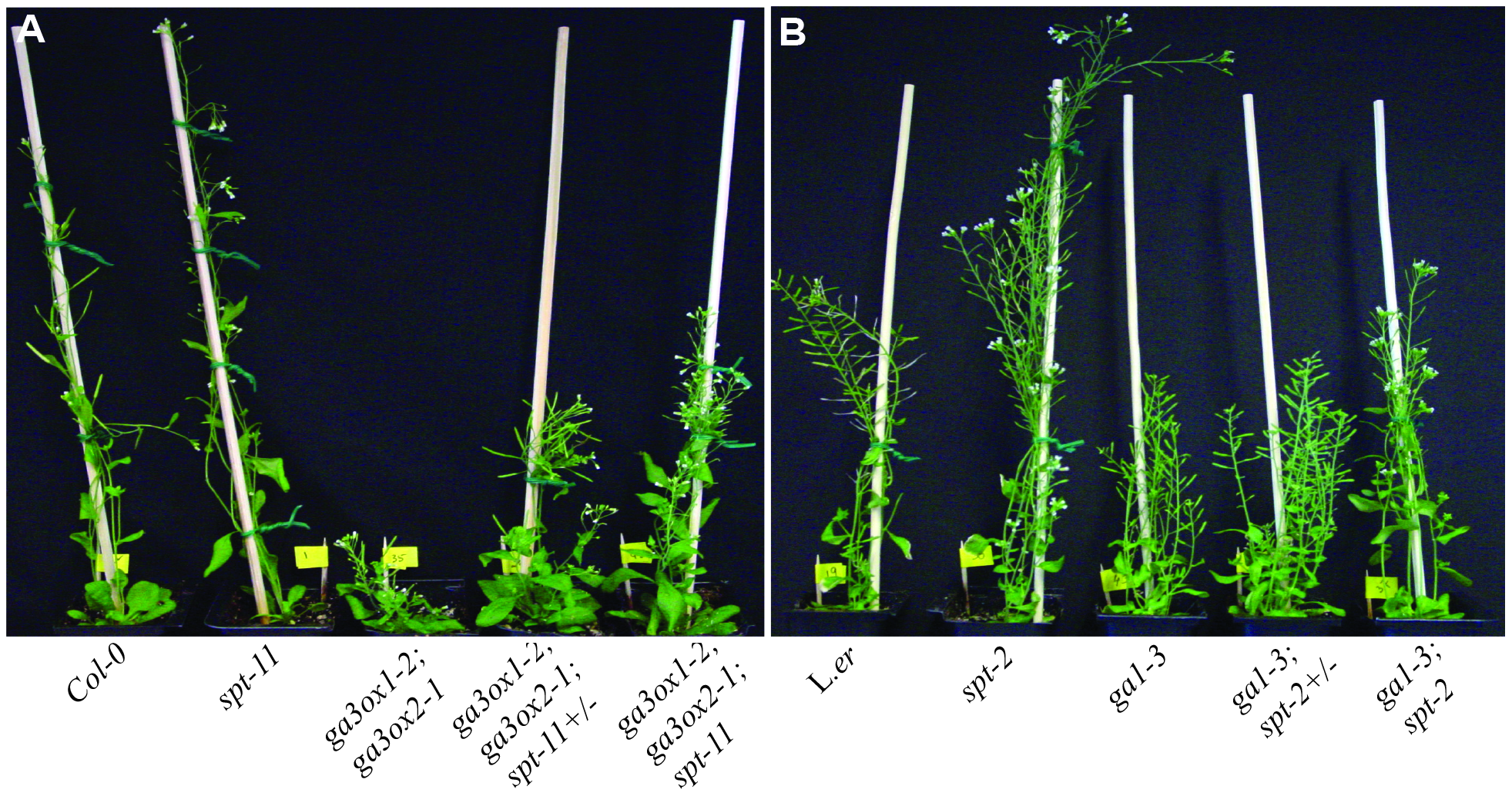

Supplement: Additional file 4 — SPTacts additively with GA. Photographs of adult plants. (A) Representative Col-0, spt-11, ga3ox1-2; ga3ox2-1, ga3ox1-2; ga3ox2-1; spt-11/+ and ga3ox1-2; ga3ox2-1; spt-11 plants. (B) Representative L. er, spt-2, ga1-3, ga1-3; spt-2/+ and ga1-3; spt-2 plants. [file 1471-2229-13-1-S4.tiff]
