# Supplementary material for: The bHLH transcription factor SPATULA regulates root growth by controlling the size of the root meristem
Source: BMC Plant Biol. 2013 Jan 2;13:1. doi: 10.1186/1471-2229-13-1 (PMC3583232; doi:10.1186/1471-2229-13-1)
Supplement: Additional file 5 — SPT and GA act additively. [file 1471-2229-13-1-S5.docx]

**Additional file 5. SPT and GA act additively^a^.**

Values are means ± standard error. ^a^All plants grown together. ^b^Measured in centimeters. ^c^Values significantly different from wild type at P<0.5. ^d^Values significantly different from *spt-11* at P<0.5. ^e^Values significantly different from *ga3ox1-2; ga3ox2-1* at P<0.05.

| **Genotype** | **Flowering time** | | **Inflorescence stem** | | | **Total plant height (cm)^b^** |
| --- | --- | --- | --- | --- | --- | --- |
|  | **Days to flower** | **Number of rosette leaves** | **Number of flowers** | **Number of internodes** | **Length of internodes (mm)^b^** |  |
| Col-0 | 23.7±0.5 | 4.5±0.2 | 49.6±1.1 | 55.6±1.4 | 0.71±0.02 | 39.2±0.9 |
| *spt-11* | 22.4±0.7 | 4.1±0.1 | 68.5±2.3^c^ | 75.4±2.2^c^ | 0.68±0.02 | 51.1±1.0^c^ |
| *ga3ox1-2; ga3ox2-1* | 24.4±0.5 | 4.5±0.2 | 42.9±2.0^c,d^ | 47.9±2.0^c,d^ | 0.25±0.02^c,d^ | 11.8±0.3^c,d^ |
| *ga3ox1-2; ga3ox2-1; spt-11* | 22.4±0.3 | 4.1±0.1 | 50.6±0.8^d,e^ | 57.1±0.8^d,e^ | 0.36±0.01^c,d,e^ | 20.8±0.6^c,d,e^ |
