# Supplementary material for: The bHLH transcription factor SPATULA regulates root growth by controlling the size of the root meristem
Source: BMC Plant Biol. 2013 Jan 2;13:1. doi: 10.1186/1471-2229-13-1 (PMC3583232; doi:10.1186/1471-2229-13-1)
Supplement: Additional file 6 — Marker lines used in this study. [file 1471-2229-13-1-S6.docx]

**Additional file 6.** Marker lines used in this study.

| Marker Line | Reference | Source |
| --- | --- | --- |
| *pCyclin B1;1::CycB1;1-GUS* | [[1](#_ENREF_1)] | Dr. Peter Doerner, University of Edinburgh |
| *QC25* | [[2](#_ENREF_2)] | Dr. B. Scheres, University of Utrecht |
| *Q1630::GFP* | [[3](#_ENREF_3)] | ABRC |
| *pPIN4::PIN4-GFP* | [[4](#_ENREF_4)] | Dr. Jiri Friml, Ghent University |
| *DR5::GUS* | [[5](#_ENREF_5)] | ABRC |
| *pSCR::GFP* | [[6](#_ENREF_6)] | Dr. Philip Benfey, Duke University |
| *pSHR::GFP* | [[7](#_ENREF_7)] | Dr. Philip Benfey, Duke University |
| *pSHR::SHR-GFP* | [[8](#_ENREF_8)] | Dr. Philip Benfey, Duke University |
| *J0121::GFP* | [[3](#_ENREF_3)] | ABRC |
| *CoYMV::GFP* | [[9](#_ENREF_9)] | Dr. Biao Ding, The Ohio State University |

**REFERENCES**

1. Colon-Carmona A, You R, Haimovitch-Gal T, Doerner P: **Technical advance: spatio-temporal analysis of mitotic activity with a labile cyclin-GUS fusion protein**. *Plant J* 1999, **20**(4):503-508.

2. Bechtold N, Ellis J, Pelletier G: ***In planta* Agrobacterium mediated gene transfer by infiltration of adult Arabidopsis plants.** *C R Acad Sci Ser III Sci Vie* 1993, **316**:1194-1199.

3. Ohashi-Ito K, Bergmann DC: **Regulation of the Arabidopsis root vascular initial population by *LONESOME HIGHWAY***. *Development* 2007, **134**(16):2959-2968.

4. Friml J, Benkova E, Blilou I, Wisniewska J, Hamann T, Ljung K, Woody S, Sandberg G, Scheres B, Jurgens G *et al*: **AtPIN4 mediates sink-driven auxin gradients and root patterning in Arabidopsis**. *Cell* 2002, **108**(5):661-673.

5. Friml J, Vieten A, Sauer M, Weijers D, Schwarz H, Hamann T, Offringa R, Jurgens G: **Efflux-dependent auxin gradients establish the apical-basal axis of Arabidopsis**. *Nature* 2003, **426**(6963):147-153.

6. Wysocka-Diller JW, Helariutta Y, Fukaki H, Malamy JE, Benfey PN: **Molecular analysis of *SCARECROW* function reveals a radial patterning mechanism common to root and shoot**. *Development* 2000, **127**(3):595-603.

7. Helariutta Y, Fukaki H, Wysocka-Diller J, Nakajima K, Jung J, Sena G, Hauser M-T, Benfey PN: **The *SHORT ROOT* gene controls radial patterning of the Arabidopsis root through radial signaling.** *Cell* 2000, **101**:555-567.

8. Nakajima K, Sena G, Nawy T, Benfey PN: **Intercellular movement of the putative transcription factor SHR in root patterning**. *Nature* 2001, **413**(6853):307-311.

9. Matsuda Y, Liang G, Zhu Y, Ma F, Nelson RS, Ding B: **The Commelina yellow mottle virus promoter drives companion-cell-specific gene expression in multiple organs of transgenic tobacco**. *Protoplasma* 2002, **220**(1-2):51-58.
