# Supplementary material for: The bHLH transcription factor SPATULA regulates root growth by controlling the size of the root meristem
Source: BMC Plant Biol. 2013 Jan 2;13:1. doi: 10.1186/1471-2229-13-1 (PMC3583232; doi:10.1186/1471-2229-13-1)
Supplement: Additional file 7 — Primers used in this study. [file 1471-2229-13-1-S7.docx]

**Additional file 7.** Primers used in this study.

| Primer name | Primer sequence 5’ to 3’ | Reference |
| --- | --- | --- |
| SMSPTF | CACCATGATATCACAGAGAGAAGAAAGAGAAGAGAAGAAG | This study |
| SPTRSTOP | TTAAGTAATTCGATCTTTTAGGTCAGGTTGTCCATC | This study |
| ACTINF | GGCGATGAAGCTCAATCCAAA | [[1](#_ENREF_1)] |
| ACTINR | GGTCACGACCAGCAAGATCAA | [[1](#_ENREF_1)] |
| spt-2dCAPSF | CCTCTTCCAAGTCAGGTCCTTCTTCTC | This study |
| spt-2sap1 | GCTTTCATTTTTTCATTAATTCTACTGCTC | This study |
| WiscdsloxsptF | GCAACTAATGTATTCTTGTTTATCACGCCAC | This study |
| WiscdsloxsptR | AGATGAACTACGAGAAGAAGGACCTGACTT | This study |
| Ds-P745 | AACGTCCGCAATGTGTTATTAAGTTGTC | [[2](#_ENREF_2)] |
| ga1-3F | TCCGAACGGTAGTACTTCATTCAATAACA | This study |
| ga1-3R | TTTCTTCATACCACCTGCGTTCAAATAC | This study |
| ga1-3 2F | CCAGAAGCGGTTCCATACATTGTTCAA | This study |
| ga1-3 2R | CGGAGAATCGTACGGTACATCAATGT | This study |
| ga3ox1F | GTGTTTAGAGGCCATCCCATTCACCT | This study |
| ga3ox1R | GAGGACAAACAAATCATATTGCTGAAATCAT | This study |
| ga3ox2F | GCCTTTTAGCATGAGTTCAACGTTGAG | This study |
| ga3ox2R | GAGAGATCATTATATCGGATGGTGGACC | This study |
| LBa1 | TGGTTCACGTAGTGGGCCATCG | [[3](#_ENREF_3)] |
| At1g13320 QF | TAACGTGGCCAAAATGATGC | This study |
| At1g13320 QR | GTTCTCCACAACCGCTTGGT | This study |
| At4g33380 QF | TCAAGCATGAGGTTTCCACTGC | This study |
| At4g33380 QR | GCGCCACGTTTCTACAACTTCTC | This study |
| GID1A QF | ATCTTATTGAGAGCAGAACAGTGGTT | This study |
| GID1A QR | TAAGTGTCGGTTAAAGGTTCCATCAG | This study |
| AtGID1b QF | CCTTAACGAATGCAAGAGAATTGTC | This study |
| AtGID1b QR | GGCGGGAACTTTACGGTCAA | This study |
| GA3 OX1 Q RT F | CCTCAACTACTGCGATATCGTTGAA | This study |
| GA3 OX1 Q RT R | AAGTGAATTTAGTGCTAACCACATCAAT | This study |
| ATGA3OX2 2QF | CCAGCCACCACCTCAAATACTGTG | This study |
| ATGA3OX2 2QR | TGCGAACCACATCAACTTGGC | This study |
| GA20 OX1 2QF | AGATTACTTCTGCGATGCGTTGG | This study |
| GA20 OX1 2QR | TCTTGATACACCTTCCCAAATGGC | This study |
| GA20 OX2 QF | CGAGCAGTTTGGGAAGGTGTATC | This study |
| GA20 OX2 QR | AATCCTCGGAAATAGTCTCGGTTT | This study |
| GA2 OX1 QF | CAGGTGATGACAAATGGGAGGTT | This study |
| GA2 OX1 QR | CAACGGAGCGATTCTCTGAGTCA | This study |
| GA2 OX2 QF | CGTGAGTCGGTGGAGGAGTACA | This study |
| ATGA3OX2 2QR | TGCGAACCACATCAACTTGGC | This study |
| GA20 OX1 2QF | AGATTACTTCTGCGATGCGTTGG | This study |
| GA20 OX1 2QR | TCTTGATACACCTTCCCAAATGGC | This study |
| GA20 OX2 QF | CGAGCAGTTTGGGAAGGTGTATC | This study |
| GA20 OX2 QR | AATCCTCGGAAATAGTCTCGGTTT | This study |
| GA2 OX1 QF | CAGGTGATGACAAATGGGAGGTT | This study |
| GA2 OX1 QR | CAACGGAGCGATTCTCTGAGTCA | This study |
| GA2 OX2 QF | CGTGAGTCGGTGGAGGAGTACA | This study |
| GA2 OX2 QR | TTCGGCAACCATCTCCAACAC | This study |
| GA2 OX4 QF | ACAGGTGATGACGAACGGGAGAT | This study |
| GA2 OX4 QR | CGTCATAACCATCGCCGAAAGA | This study |
| GA2 OX8 2QF | TGGTGACTTATTTCAGGCATGGAG | This study |
| GA2 OX8 2QR | CCTTTGGGTTCGTCATCACACG | This study |
| GAI QRTF | TTCAACGGCGGTGAGGGTTATC | This study |
| GAI QRTR | TGCCAACCCAACATGAGACAGC | This study |
| SCL3 QRTF | AGAGCGGGTGCGCAGTAATTTG | This study |
| SCL3 QRTR | TCACTTCCTGCATCTCCAAGCTG | This study |
| EXP1 QRTF | TGCTACCCTTGGAGCAATGACG | This study |
| EXP1 QRTR | ACAAGCACCTCCCATTGTGC | This study |

**REFERENCES**

1. Teotia S, Lamb RS: **The paralogous genes *RADICAL-INDUCED CELL DEATH1* and *SIMILAR TO RCD ONE1* have partially redundant functions during Arabidopsis development**. *Plant Physiol* 2009, **151**(1):180-198.

2. Woody ST, Austin-Phillips S, Amasino RM, Krysan PJ: **The WiscDsLox T-DNA collection: an arabidopsis community resource generated by using an improved high-throughput T-DNA sequencing pipeline**. *J Plant Res* 2007, **120**(1):157-165.

3. Alonso JM, Stepanova AN, Leisse TJ, Kim CJ, Chen H, Shinn P, *et al*: **Genome-wide insertional mutagenesis of Arabidopsis thaliana**. *Science* 2003, **301**(5633):653-657.
