# Supplementary material for: The bHLH transcription factor SPATULA regulates root growth by controlling the size of the root meristem
Source: BMC Plant Biol. 2013 Jan 2;13:1. doi: 10.1186/1471-2229-13-1 (PMC3583232; doi:10.1186/1471-2229-13-1)
Supplement: Additional file 8 — Primer combinations used for genotyping. [file 1471-2229-13-1-S8.docx]

**Additional file 8.** Primer combinations used for genotyping.

| Allele | Wild type specific Primers | Mutant specific primers |
| --- | --- | --- |
| *spt-2* | spt-2dCAPSF and spt-2Sap1 | spt-2dCAPSF and spt-2Sap1 |
| *spt-11* | WiscdsloxsptF and WiscdsloxsptR | WiscdsloxsptF and Ds-P745 |
| *ga1-3* | ga1-3 2F and ga1-3 2R | ga1-3F and ga1-3R |
| *ga3 ox1-3* | ga3ox1F and ga3ox1R | ga3ox1R and LBa1 |
| *ga3 ox2-1* | ga3ox2F and ga3ox2R | ga3ox2R and LBa1 |
