# Supplementary material for: The bHLH transcription factor SPATULA regulates root growth by controlling the size of the root meristem
Source: BMC Plant Biol. 2013 Jan 2;13:1. doi: 10.1186/1471-2229-13-1 (PMC3583232; doi:10.1186/1471-2229-13-1)
Supplement: Additional file 9 — Primer combinations used in qRT-PCR. [file 1471-2229-13-1-S9.docx]

**Additional file 9.** Primers used in qPCR.

| Gene | Primer pair | Product size (base pairs) |
| --- | --- | --- |
| *SPATULA* | SPT Q RTF and SPT Q RTR | 76 bps |
| *GA3 OX1* | GA3OX1 Q RT F and GA3OX1 Q RT F | 88 |
| *GA3 OX2* | ATGA3OX2 2QF and ATGA3OX2 2QR | 86 |
| *GA20 OX1* | GA20 OX1 2QF and GA20 OX1 2QR | 60 |
| *GA20 OX2* | GA20 OX2 QF and GA20 OX2 QR | 117 |
| *At GID1a* | GID1A QF and GID1A QR | 113 |
| *At GID1b* | AtGID1b QF and AtGID1b QR | 142 |
| *GA2 OX1* | GA2 OX1 QF and GA2 OX1 QR | 123 |
| *GA2 OX2* | GA2 OX2 QF and GA2 OX2 QF | 72 |
| *GA2 OX4* | GA2 OX4 QF and GA2 OX4 QR | 142 |
| *GA2 OX8* | GA2 OX8 2QF and GA2 OX8 2QR | 74 |
| *At1g23205* | At1g23205 2QF and At1g23205 2QF | 73 |
| *At2g21220* | At2g21220 2QF and At2g21220 2QR | 71 |
| *At2g45210* | At2g45210 2QF and At2g45210 2QR | 80 |
| Reference genes | |  |
| *At1g13320* | At1g13320 QF and At1g13320 QR | 61 |
| *At4g33380* | At4g33380 QF and At4g33380 QR | 149 |
